# The experiences, needs and barriers of people with impairments related to usability and accessibility of digital health solutions, levels of involvement in the design process and strategies for participatory and universal design: a scoping review

**DOI:** 10.1186/s12889-021-12393-1

**Published:** 2022-01-06

**Authors:** Silje Havrevold Henni, Sigurd Maurud, Kristin Skeide Fuglerud, Anne Moen

**Affiliations:** 1 Department of Nursing Science, Institute of Health and Society, Faculty of Medicine, University of Oslo, P.O. Box 1130, Blindern, NO-0318 Oslo, Norway; 2 Norwegian Computing Center, P.O. Box 114, Blindern, N-0314 Oslo, Norway

**Keywords:** Disability, Digital health, Health services accessibility, User participation, Participatory design, Universal design, Inclusive design

## Abstract

**Objective:**

Globally, the number of digital health solutions is increasing, but they are not always designed with access and utilisation for people with impairments in mind. Development efforts have often not included the voice and requirements of people with impairments, who make up 15% of the world’s population, despite the fact that this can help ensure broad access and utilisation. Little attention to and limited inclusion of people with impairments in the development of digital health solutions results in continued and reinforced inequalities in health services provision for people with impairments. This review investigates the needs and barriers of people with impairments related to use of digital health solutions and strategies to foster user participation, access and utilisation of digital health solutions.

**Methods:**

This scoping review, based on the Joanna Briggs Institute Manual, had five phases: 1) identification of aim and research questions, 2) literature search in five databases (April/May 2020), 3) literature screening based on predetermined inclusion and exclusion criteria, 4) data extraction, and (5) reporting results.

**Results:**

The literature search resulted in 5968 sources, of which 25 met our inclusion criteria. People with impairments appreciate digital health solutions that are designed to meet their specific impairment-related challenges. The reported needs and barriers related to technological design varied depending on the individuals’ challenges. The literature reported different types of participatory co-design strategies to foster access and utilisation of digital health solutions.

**Conclusion:**

This scoping review support needs for increased awareness among developers to design solutions that meet people’s needs, contexts and states of health. By applying universal design as a strategy and including people with different types of impairments, starting in the idea creation phase of digital health solutions and throughout the development, developers can design solutions with better accessibility. Digital health solutions that are accessible and usable have a tremendous opportunity to foster health equity and achieve health promotion, prevention and self-care. This in turn can contribute to closing the gap between different population groups, reduce disparities and get the most from available healthcare services.

**Supplementary Information:**

The online version contains supplementary material available at 10.1186/s12889-021-12393-1.

## Introduction

Globally, a plethora of digital health solutions has been suggested, designed and/or deployed. During COVID-19, many countries adopted social distancing measures, and the availability of and access to digital health solutions for all are therefore highly pertinent to ensure good public health. Digital health is a broad term that includes the use of information and communication technology, such as software applications, mobile phones and wearable devices, to support peoples’ health and their quality of life. The World Health Organization (WHO) emphasises that digital health is essential in achieving universal health coverage as “it extends the scope, transparency and accessibility of health services and health information, widening the population base capable of accessing the available health services and offering innovation and efficiency gains in the provision of health care” [1]. In this paper we focus on digital health solutions and services intended for personal use. Examples are apps and devices for self-management and monitoring of health conditions and contact with health professionals.

People with impairments (loss or abnormality of body functions, sensory and cognitive capacities, and structures) can often experience barriers leading to de-facto disabilities due to the structure of society [2], and digital health solutions are part of such an exclusive structure if they are not designed to be accessible to people with impairments. According to WHO and the World Bank, people who experience disabilities make up 15% of the world’s population [2]. The term disability has had different meanings throughout the ages due to public policy [3]. Disability is in this article defined according to the International Classification of Functioning, Disability and Health (ICF) [4]. The ICF is structured around three components: 1) body functions and structures (physiological, psychological and anatomical), 2) activities (the execution of a task or action by an individual) and participation (involvement in a life situation), and 3) environmental (the specific context) and personal factors (lifestyle, social background) that may have an impact on the individual’s health and health-related states. According to ICF, a disability occurs when there is a limitation in activity and participation because of a gap between an individual’s body function and structure, and personal experiences and knowledge on the one side, and the requirements from the environment on the other side. The ICF definition indicates that there is a correlation between disability and disadvantage and that the level of disadvantage depends on the gap between the factors described above.

In this article, we have chosen to use the term impairment for the population group we study, as impairment does not lead to or justify disability. It is rather society’s lack of consideration of the needs of people with impairments that leads to disability. People can be born with an impairment, acquire an impairment as a result of an accident or injury, or develop an impairment because of chronic conditions or harsh environmental conditions. We adopted a disease-agnostic perspective, which means that we will focus on functional impairments (cognitive, mental, motor, visual and hearing) regardless of what circumstance caused the impairment or disease. The decision to apply this perspective reflects our assumption that people with similar functional impairments may have similar experiences independent of the cause of their impairments. Furthermore, a disease-agnostic perspective for this scoping review may provide insights that will be valuable for the design for and collaboration with people with different types of impairments independent of the cause of impairment.

Accessibility problems of digital health occur in the intersection between the user, their context and the product or service in question. People with impairments have challenges related to digital health solutions, if development processes fail to consider the diversity of the human body function and structure, their abilities and their context. For example, visually impaired people need descriptions of images, while people with hearing impairment need captioning of videos, people with loss of hand dexterity may need to give input through voice recognition, people with motor impairments may need their health applications to be able to measure activity when using a mobility aid, and people with cognitive impairments may need an interface with easily recognisable and familiar icons and text to speech functionality [5]. Developers of digital health solutions should therefore strive to develop solutions that are universally designed, which means that they are accessible and usable for everyone, including people with impairments. For example, an app for measuring blood sugar for people with diabetes should be designed so that it can be used by people with visual impairments or other types of impairments, as people needing to measure their blood-sugar may have different impairments independent of their diabetes.

To make digital health solutions more accessible and usable to people with a diversity of body function and structure, developers of digital health solutions should conform to the Web Content Accessibility Guidelines (WCAG) [5]. These guidelines have been specially developed with the aim of making web content more accessible to people with impairments, but also for people in diverse contexts and with a range of user devices, including smart phones. The WCAG guidelines are widely accepted and referred to in legislation, such as the EU Directive on the accessibility of websites and mobile applications. They are also integrated into the US Section 508 of the Rehabilitation Act of 1973. However, technical reviews show that health websites and applications in general do not conform to these guidelines [6–8]. This is also confirmed by people with impairments who report difficulties with accessibility and usability of digital health solutions [9–14].

People with impairments often experience disability when they are exposed to inequality, disparity, discrimination and systemic exclusion. David ( [15] , p. 253) explains that when it comes to “race it is not the skin that matters, but rather the meanings ascribed to that skin by ideology, and the consequent jaundiced ways in which society responds to its ‘inhabitants’. So, too, in disability”. For example, women with different types of impairments are systematically excluded from traditional preventive health screenings like mammography [16], and university students who use wheelchairs can have problems pursuing higher education due to lack of access to auditoriums, toilets, libraries and transport facilities [17]. Discrimination and systemic exclusion of people with impairments has resulted in the establishment of the Convention on the Rights of Persons with Disabilities (CRPD) in 2007 [18]. The purpose of the Convention is to ensure human rights for people with impairments who experience disabilities. Both the right to the highest attainable standard of health (Article 25) and information technology (Article 19) is specified in the Convention. According to the articles, digital health solutions should be accessible and usable for people with impairments, and universal design is the recommended strategy to achieve this goal.

Inclusion of people with different types of impairments in the development cycle of digital health solutions may lead to design of solutions with better accessibility experiences for the end users. Ideal participation would be to involve people with different types of impairments early in the idea creation phase of digital health solutions and throughout the development [19]. However, there is lack of active inclusion of the voice and requirements by people with impairments in health research in general, despite several published studies for how to include people with impairments in research [20–24] and the guidance of CRPD [18]. Limited inclusion of people with impairments in the development of digital health solutions easily lead to continuation and reinforcement of inequalities in health services provision for people with impairments. A preliminary search for literature on digital health solutions and people with impairments in PubMed, CINAHL, EMBASE and Cochrane showed that people with impairments are rarely included in research about digital health solutions, not even when the solution is specifically designed to be used by people with impairments. This contrasts with the clearly stated need for digital health solutions that are suitable, accessible and usable for people with impairments [9, 10, 14].

### Aim

The aim of this scoping review is to investigate the needs and barriers of people with impairments related to the use of digital health solutions, and strategies to foster user participation, access and utilisation of digital health solutions. The scoping review was guided by the following research questions:

1. What needs and barriers do people with impairments experience related to the use of digital health solutions?
2. What are the levels of participation of people with impairments in the idea creation, design and evaluation phases of the design process?
3. What strategies have been suggested, implemented or evaluated to foster user participation in the design of digital health solutions for people with impairments?

## Methods

The methodology for this scoping review was based on Joanna Briggs Institute Manual [25], and had five phases: 1) identify aim and research questions, 2) search for relevant literature, 3) literature screening and selection, 4) data extraction, and (5) summarise and report the results. The title, aim, research questions, screening process and inclusion and exclusion criteria for inclusion of literature were specified in a protocol written in Norwegian before we started searching for literature. We used the PRISMA-ScR guidelines in reporting this study (see Appendix 1).

### Search strategy

In April/May 2020, with the guidance of a medical librarian we searched the following databases for relevant literature: Medline, CINAHL, Scopus, IEE Explore and ACM library. In addition, KSF hand searched The Journal on Technology and Persons with Disabilities. The databases were searched via a search query using the PCC framework [25]:

- Population – people with functional impairments (cognitive, mental, motor, visual and hearing)
- Concept – digital health solutions
- Context – user participation explained as engagement in design, access and utilisation

Appendix 2 shows the full search conducted in the different databases and Appendix 3 shows the identified literature from the databases. To confirm the quality of our search, we also searched Google Scholar with the following keywords “disability and digital and participatory design” (17,500 hits) and screened the first 50 hits, sorted by relevance, to ensure that all sources that seemed relevant to our research questions were included in the database search.

### Literature – screening and selection

All the literature was screened in two iterations by using Rayyan which is a tool developed for screening of literature [26]. Each source found in the databases was screened based on predetermined inclusion and exclusion criteria (Table 1). In the first iteration the screeners included or excluded literature based on title and abstract examination and the second iteration was based on full-text examination. In addition, the screeners followed citation trails of relevant studies.

**Table 1:** Inclusion and exclusion criteria for literature

Inclusion criteria	Exclusion criteria
Literature published between 2015-2020^a^ Refereed journal articles Full text conference papers Editorials	Literature published before 2015 Literature published that is NOT Refereed journal articles Full text conference papers Editorials
Literature published in English, Norwegian, Danish and Swedish	Literature published in other languages than English, Norwegian, Danish and Swedish
Literature that discusses people with functional impairments (cognitive, mental, motor, visual and hearing)	Literature in which the participants are health care professionals or other caregivers for people with impairments
Literature that discusses digital health solutions	Literature that discusses digital solutions that is not related to health
Literature that discusses user participation related to participation in design, access or utilisation	Literature that discusses user participation in general but does not relate it to digital health solutions
Literature that clearly addresses the perspective of people with impairments	Literature that has not addressed the perspective of people with impairments Literature where the focus is on the underlying cause of the impairment (disease) rather than on the impairment itself

^a^The time frame of 5 years was chosen due to the recent and rapid development of digital health solutions

In the first screening round, SHH and SM individually screened all the literature identified in the literature search. Prior to embarking on literature selection, SHH, SM and AM pilot screened a sample of 25 titles/abstracts to achieve consensus on literature selection. After the first screening SHH and SM disagreed upon inclusion/exclusion on 150 sources (3,6% of the sources screened). SHH, SM and AM met to discuss these sources and agreed on which sources to include. Thereafter, AM checked a random sample of the included to ensure they met the inclusion criteria. In the second screening round, SHH and SM individually screened all the sources included in the first screening round and agreed on which sources to include based on reading the full-text. We did not conduct a quality assessment of the literature as this it is not a priority in scoping reviews [27].

### Data extraction

A data extraction form was developed which included the following extraction fields:

- Study characteristics (author, year, country, aim, design, sample size)
- Population characteristics (gender, age, type of impairment)
- Key findings that relate to the scoping review questions
- Use of universal design guidelines such as WCAG

During the data extraction SHH and SM independently read the full text sources to complete the form. Through this process, SHH and SM read specifically to identify all aspects of barriers to use, access and utilisation, levels of user participation and engagement in the design process, and strategies for participatory and universal design. Most data about user participation were extracted from the method section of the included papers, while the data about needs and barriers related to design, access and utilisation of digital health solutions were extracted from the result section. As the review was disease-agnostic, what is presented in the extraction form are not the key results of the selected papers, but rather a description of people with impairments’ perceptions related to use, access and utilisation of digital health solutions, their participation in the research process, and strategies for participation and universal design. All the extracted data are presented in tables and summarised in the result section.

## Results

### Literature search

**Fig. 1:**
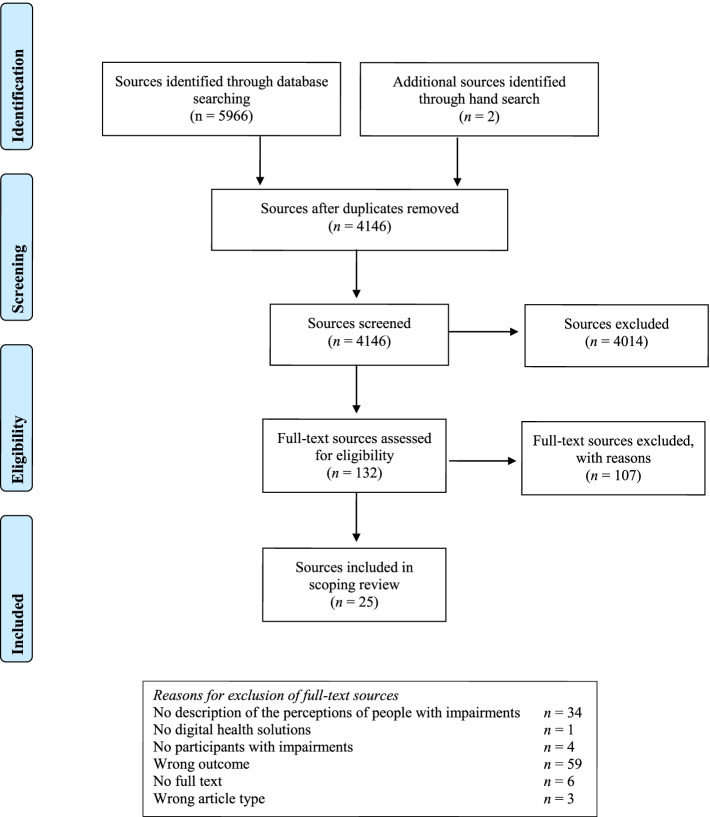
PRISMA Flow Diagram

The literature search resulted in 5968 sources before removal of 1822 duplicates. In the first screening round, a large proportion of the sources were excluded due to outcomes with another focus than this study, population and type of publication, lack of user involvement and a focus on digital solutions that are not related to health. Another significant number of sources were randomised controlled trials, pre-post-test studies or similar comparison designs, which focused on whether digital health solutions have the desired effect. These sources were also excluded as they did not report the perspectives of people with impairments, barriers, access and utilisation of the digital health solution. In the second round of screening, it became clear that several of the sources reported participatory design in the development of digital health solutions as a strategy to foster access and use of the solution, however they did not report how the perspective of people with impairments were attended to during the development process; from idea to design and deployment. A large proportion of the literature was therefore excluded in the second round of screening as we only included studies that reported how the perspectives of people with impairments were incorporated into the development process. In addition, several sources were excluded because they only focused on the technical feasibility of the digital health solution. Finally, a total of 25 sources were included in the study. The screening process is summarised in Fig. 1.

### Characteristics of included literature

Table 2 shows the characteristics of the included literature. A large proportion of the sources reported different types of apps that can be used on smart-phones and tablets [28–42]. The other sources included a smart shower [43], conversational agents [44], gaming, social media, and robotics technologies [45], website [46], interactive map [47], augmented reality magnification aid device for low vision user [48], electric powered wheelchair [49] and strategies for how to design digital health solutions [50–52]. An overview of the included literature is provided in Table 3.

**Table 2:** Overview of the characteristic of the included sources (*n* = 25)

Variables	Sources (*n* = 25)
Countries	
Australia	1
Canada	2
Colombia	1
France	1
India	1
Italy	1
Netherlands	1
South Korea	1
Sweden	2
Taiwan	1
United Kingdom (UK)	2
United States of America (USA)	11
Publication year	
2015	2
2016	2
2017	4
2018	11
2019	3
2020	3
Type of impairment (some sources included more than one impairment)	
Cognitive impairment	10
Motor impairment	8
Visual impairment	7
Hearing impairment	2

**Table 3:** Overview of the included sources (*n* = 25)

Author, year and country	Aim	Design^a^	Sample	Type of impairment	Key findings that relate to the scoping review questions
Albouys-Perrois, Laviole et al. [47] – 2018, France	To design an accessible interactive map, using a participatory design approach.	Participatory design, includes participants in the design phase	15 visually impaired students (6 females and 9 males, age range 11–40), 3 orientation and mobility instructors, 22 special education teachers and orientation and mobility instructors, 1 orthoptist, 2 tactile transcribers, and 1 technical advisor (unknown characteristics)	Visual impairment	Using a participatory design approach, the researchers have designed an augmented reality map based on the needs of people with visual impairment. A usability test shows that the participants evaluated the usability of the solution to be good.
Arnott, Malone et al. [52] – 2018, UK	To present strategies to adapt research processes to suit people with cognitive and communication impairments when developing smartphone apps to give access to health promotion information.	Unknown	Unknown	Cognitive and speech impairment	To involve participants with cognitive impairments in the development process, the researchers implemented strategies to make information more accessible to the participants. This included simplified language at all levels of the research process, alternative forms of language representation, and the use of an app to enable participants to experience unfamiliar technology.
Bhattacharjya, Stafford et al. [39] – 2019, USA	To create technology that supports individuals with sensorimotor deficits self-manage upper limb rehabilitation at home.	Experimental design, includes participants in the idea creation phase	4 older adults (gender unknown, age range 66–87) and 4 individuals with stroke (gender unknown, age range 54–68)	Motor impairment	Usability ratings from the participants led the researchers to modify the design of the 3D printed items and improve the clarity of the mRehab app.
Blair and Abdullah [44] – 2019, USA	To understand how and why people with hearing impairments use conversational agents.	Qualitative design (interview)	4 adults with hearing impairment (3 females and 1 male, age range 53–63)	Hearing impairment	Female voices are default for most solutions, but these high-pitched voices are often hard to hear with hearing aids or cochlear implants. The speed of the participants’ conversational agents was often too fast, so they had to repeat the message.
Daveler, Salatin et al. [49] – 2015, USA	To understand the conditions and barriers electric powered wheelchair (EPW) users find difficult to drive in/over in the outdoor environment (phase I), create a computer-aided design prototype of an EPW with advanced features that increase the users’ safety and ease navigation when encountering such conditions and barriers (phase II), and validate the newly designed EPW and its advanced features by gathering further input from EPW users (phase III).	Participatory design, includes participants in the idea creation phase	Phase I: 31 EPW users (5 females, 26 males, average age 55.8) Phase III: 12 EPW users (5 females, 7 males, average age 46.9)	Motor impairment	The researchers have designed an EPW based on EPW users’ needs. The designed EPW received positive feedback from the users.
DeForte, Sezgin et al. [28] – 2020, USA	To evaluate the user experience of the Hear Me Read app.	Qualitative design (focus group), includes participants in the evaluation phase	8 children with hearing impairment (4 females and 4 males, age range 2–13), 8 caregivers (unknown characteristics)	Hearing impairment	The children found the swiping motion hard to use, so they had difficulties navigating the app. Children instinctively pressed a single point to navigate.
Ferati, Babar et al. [43] – 2018, Sweden	To identify the type of smart product that mostly increases people with impairments independence at home.	Participatory design, includes participants in the idea creation phase	6 adults with different types of impairment (3 females and 3 males, age range 20–87)	Motor, visual and cognitive impairment	The results showed that the participants needed a smart shower. The buttons on the smart shower were designed to have distinctive shape, colour, and dimension to be accessible and usable by people with visual impairment. The researchers describe that involvement of participants in identifying the need helped them to understand the participants’ ways of doing things in their homes. Cartographic mapping was useful to understand the differences among the participants, even those with the same impairment. This indicates that people with similar impairments should not be treated homogeneously as their wishes and issues might differ.
Furberg, Ortiz et al. [41] – 2018, USA	To describe the design and development process of a tablet-based decision support tool to enhance shared decision making and decisional capacity for those with Fragile X Syndrome participating in the informed consent process.	User-centred design, includes participants in the idea creation phase	6 adults with Fragile X syndrome (1 female and 5 males, age range 16–28), 3 clinician stakeholders (unknown characteristics)	Cognitive impairment	Even though the participants were included in the idea creation process, the perspectives of the persons with Fragile X Syndrome are in lesser degree described. The researchers highlight the importance of a user-centered approach, appreciation of interdisciplinarity, and stakeholder engagement and input as central key findings in the design and development process of this decision support tool.
Glaser, Schmidt et al. [46] – 2017, USA	To design, develop, and evaluate the Epilepsy Journey intervention for adolescents with epilepsy and executive functioning deficits at a Midwestern epilepsy centre.	Participatory action research, includes participants in the idea creation phase	11 adolescents with epilepsy (gender unknown, age range 13–17), 5 primary caregivers and 1 usability expert (unknown characteristics)	Cognitive impairment	The results provide instructional designers insight into how a user-centric formative design approach can be used for the development of individually tailored web-based interventions for sensitive populations.
Groussard, Pigot et al. [40] – 2018, Canada	To design an assistive device, SAMI (Services Assistance Mobile and Intelligent), to improve the social participation of people with traumatic brain injury, and to conduct a proof of concept to show how this assistive device fulfilled the needs of some people with traumatic brain injury.	Participatory design, includes participants in the idea creation phase	4 adults with head injury (4 males, age range 30–70), 3 caregivers (unknown characteristics)	Cognitive impairment	The process was adapted to propose tangible and short activities that do not challenge memory and abstraction. Focus groups solicited active participation by gathering reviews on existing applications. The votes on the various solutions proposed were a good way to obtain engagement from all the participants. This process enabled involvement of the people with cognitive impairments and their caregivers during all the development phases.
Hill and Breslin [29] – 2016, Australia	To explore the usability and acceptability of an app from the perspective of participants with aphasia and speech-language pathologists.	End-user design, includes participants in the evaluation phase	5 adults with aphasia (1 female and 4 males, age range 67–78), 3 speech-language pathologists (3 females, unknown age range)	Communication impairment	Barriers to use: scroll-bare, the one-screen keyboard, the tablets responsiveness to touch and limited previous experience with use of tablets or PC. Facilitators to use: the large screen size and support to access and use of the system.
Kerkhof, Bergsma et al. [30] – 2017, Netherlands	To explore what people with dementia find important in their choice and use of apps.	Qualitative exploratory design	8 people with dementia (2 females and 6 males, age range 60–82,), 10 informal carers (6 females and 4 males, age range 62–79)	Cognitive impairment	The people with dementia encountered several problems in navigating the apps (unclear symbols for buttons, sensitivity of the touchscreen, use of links, updates). They mentioned lay-out features that make apps attractive to use: the use of clear pictures and photos, readable letter types and sizes, a calm interface and background, contrast between text and background. Foreign language can be a barrier.
Kim, Kim et al. [51] – 2018, South Korea	To analyse the compliance rate of 120 health apps from 12 countries with the UX design guidelines and to conduct an experiment to examine whether the UX design guidelines improve the accessibility to visual information.	Experimental design, includes participants in the evaluation phase	23 people with low vison and 23 people with no impairment (unknown characteristics)	Visual impairment	The researchers have developed guidelines to make apps accessible for people with visual impairment. The study successfully confirmed the increase in the actual information recognition speed from both test groups after applying UX design guidelines to selected health apps.
Lam, Tatla et al. [45] – 2015, Canada	To establish the current use and perceptions of gaming, social media, and robotics technologies for rehabilitative purposes from the perspective of adults and children with upper limb impairments to identify barriers and enablers to their adoption and use.	Qualitative design (focus group), includes participants in the idea creation phase	7 children with motor impairment (1 female and 6 males, age range 6–16), 8 adults with motor impairment (2 females and 6 males, age range 45–75)	Motor impairment	Based on the feedback from the study’s participants, a successful gaming system should consider the following: incur low cost, demonstrate improved recovery, be simple to operate, be space-efficient, prescribe unique exercises, offer challenging and motivating games, incorporate the unaffected limb(s), create social connections, and demonstrate a clear distinction between gaming for therapy and for leisure.
Lazar, Woglom et al. [42] – 2018, USA	To describe the user-centred design process of developing a smart phone app that can potentially help people with Down syndrome make better nutritional decisions when dining out at a restaurant.	User-centred design, includes participants in the idea creation phase	10 adults with Down syndrome (5 females and 5 males, age range 18–30), caregivers (unknown number and characteristics)	Cognitive impairment	This project documents how user-centered design processes can occur for complex projects involving people with Down syndrome and potentially persuasive technology.
Leporini and Buzzi [50] – 2018, Italy	To collect blind users’ expectations and habits regarding home automation technology.	Participatory study	42 adults with visual impairment (15 females and 27 males, age range 18 < 70)	Visual impairment	Based on the to the feedback from the participants, a set of general suggestions for designers and developers of home automation and remote-control systems has been proposed to enhance accessibility and usability for the blind user.
Lu, Lin et al. [31] – 2017, Taiwan	To design an optimised and user-friendly cognitive training game for older adults.	Qualitative approach of design-based research, includes participants in the evaluation phase	9 adults (7 females and 2 males, age range 61–90), 5 stakeholders and interaction designers (2 females and 3 males, unknown age range)	All older adults receive care, 4 had cognitive impairment	The older adults responded that they preferred larger icons and words. Small icons were hard to read and tap.
Madrigal-Cadavid, Amariles et al. [35] – 2020, Colombia	To design and develop a mobile app of drug information for people with visual impairment, which allows them to access information for the appropriate use of medicine.	User-centred design, includes participants in the idea creation phase	48 adults with visual impairment (23 females and 25 males, mean age 42)	Visual impairment	A mobile app of drug information for people with visual impairment using a user-centred design process was designed and developed.
Malu and Findlater [32] – 2016, USA	To assess the accessibility of mobile and wearable fitness tracking for users with mobility impairments.	Participatory design, includes participants in the evaluation phase	14 adults with mobility impairments (7 females and 7 males, age range 22–72)	Motor impairment	Manual input of data on a mobile app is challenging. Existing solutions were not relevant to their abilities. Participants’ designs and rationale suggest that: an unobtrusive wearable form factor is best, but it needs to be easy to put on and take off; preferences related to mobility level suggest that it will be important to cater to the needs of each user.
Singanamalla, Potluri et al. [36] – 2019, India	To investigate the state of accessibility of ATM machines in India and describe low-cost design changes that could potentially improve the accessibility of these machines for visual impaired persons in India.	Mixed methods, includes participants in the idea creation phase	22 adults with visual impairment (5 females and 17 males, age range 18–50)	Visual impairment	Based on surveys with visually impaired persons the researchers developed a solution and perform a usability evaluation with the persons that participated in the survey.
Stearns, Findlater et al. [48] – 2018, USA	To investigate the design possibilities for augmented reality magnification tools enabled by registering virtual content in real 3D space.	Iterative design, includes participants in the design phase	7 adults with visual impairment (4 females and 3 males, age range 28–68)	Visual impairment	The researchers first designed a prototype of a solution. Thereafter, the solution was further developed based on design sessions with persons with visual impairment. The persons tested the solution and based on their feedback the researchers updated the solution and some of the persons then tested the solution again.
Thirumalai, Rimmer et al. [38] – 2018, USA	To describe the development process of the TEAMS (Tele-Exercise and Multiple Sclerosis) app, which is being used by people with multiple sclerosis in a large randomised controlled trial to engage in home-based tele rehabilitation.	Parallel-iterative design, includes participants in the design phase	21 adults with motor impairment (14 females and 7 males, mean age 54)	Motor impairment	Following several iterative evaluations, the project team and participants finalised an exercise app that can be easily operated in the convenience of the home and tailored to the functional needs of individuals with multiple sclerosis.
Winberg, Kylberg et al. [33] – 2017, Sweden	To describe how persons ageing with neurological disorders experience barriers and facilitators in relation to using apps in everyday life.	Qualitative design (focus group)	16 adults with neurological disease (5 females and 11 males, age range 51–74)	Fine motor skills, motor and cognitive impairment	The usability of apps was impacted by impairments in fine motor skills and sensory functions. Difficulties with fine motor skills and sensory functions made it hard to operate smartphones or tablets with two hands. The participants agreed that apps need to be easy and intuitive to use.
Zafeiridi, Paulson et al. [34] – 2018, UK	To explore the usability and user-friendliness of the CAREGIVERSPRO-MMD platform through evaluations performed by people with dementia or mild cognitive impairment, informal caregivers, and health and social care professionals.	Mixed methods design, includes participants in the evaluation phase	24 older adults with dementia or mild cognitive impairment (14 females and 10 males, age range 55–91), 34 informal caregivers (20 females and 4 males, age range 30–77), 10 professionals (7 females and 3 males, age range 26–53)	Cognitive impairment	People with dementia preferred bigger colour contrasts and font sizes, as well as images and icons rather than text menus. Emoticons were used in the platform to like or not-like messages, but people with dementia found this confusing.
Zhou, Saptono et al. [37] – 2020, USA	To identify an approach that can be generally applied to improve the accessibility of mHealth apps.	Participatory design, includes participants in the idea creation phase	5 adults with cerebral palsy or spinal cord injury (3 females and 2 males, age range 16–41)	Motor impairment (fine motor skills)	The study participants tested an app and experienced various levels of difficulty related to font size and style, spacing, button and selection option arrangement, colour and contrast, data input, page navigation and handedness. The accessibility of the apps was improved after the participants’ desired accessibility features were added into the app.

^a^A description of when people with impairments are included in the research phase is included if available

### The needs and barriers related to the use of digital health solutions

The literature reported different needs and barriers related to the technological design. For example, people with visual impairment state that digital health solutions that include buttons must be designed to ensure that the buttons have distinctive shape, colour, and dimension to be accessible and usable [43]. People with hearing aids or cochlear implants express that most conversational agents with voice interfaces have female voices as default and that this can be a barrier, as high-pitched voices are often difficult to hear, especially if the speed of voice is too fast [44]. Furthermore, adults with motor impairment request health and fitness tracking solutions designed to cater to the mobility level and needs of each user (e.g., tracking rolling and posture) [32]. Children and adults also request rehabilitation gaming systems designed for people with motor impairment, as such games encourage users to do exercises that otherwise seem repetitive and tedious [45]. In addition, they expressed that the games should be space-efficient and have low cost for the user.

On a general basis and regardless of the type of impairment, the literature described that digital health solutions must be designed to be easy and intuitive to use [30–33, 37]. Adults with impairment in fine motor skills and sensory functions express that it is challenging to operate smartphones and tablets with two hands [33]. Manual input of data on apps is also experienced to be a barrier for those with motor impairment [32, 37]. Older adults with some cognitive impairment also express that input of data in apps should be intuitive and that the apps should have large icons and words, as small icons are hard to read and tap [31]. Furthermore, older adults with cognitive impairments express that the use of apps in general (e.g., games apps, news and weather forecast apps) can be a barrier for them as they stated that apps are often hard to navigate and use, and their capacities to comprehend is further compromised due to unclear symbols for buttons, sensitivity of the touchscreen, use of links, updates that change the operation of the app and foreign language [30]. Features promoting use of apps among older adults were carefully selected layout features, such as use of clear pictures and photos, readable letter types and sizes, a calm interface and background, and a contrast between text and background.

The authors of several of the included sources have designed and developed digital health solutions intended for people with impairments and invited people with impairments to test the usability of the solution and service [28, 29, 34, 37]. In accordance with the sources presented above, the testers reported varying degrees of challenges related to the layout and navigation of the apps, indicating that it is challenging to design a user-friendly app.

### Strategies that have been suggested, implemented or evaluated to foster user participation, access and utilisation

Overall, the literature reported use of different types of participatory design strategies to foster access and utilisation of digital health solutions for people with impairments. However, it differed where in the development process they chose to invite perspectives of people with impairments. Two of the sources reported using WCAG [5] in the development process as a strategy to foster access and utilisation of digital health solutions for people with impairments [42, 51].

All sources presented in this section report that participants with impairments were actively involved in the entire design process. Adults with visual [35, 36, 47], motor [37, 49], and cognitive ([40, 41, 46] (adolescents)) impairment were included as participants in the idea creation process to identify the needs of the end-users. Then the researchers developed a digital health solution that the participants contributed to test and evaluate for usability and accessibility. The participants expressed that the accessibility and usability of the solution is good [35–37, 40, 41, 46, 47, 49]. Lazar, Woglom et al. [42] also included adults with cognitive/intellectual impairment in the idea creation process to identify the needs of the end-users. Despite a thorough description of how people with cognitive/intellectual impairment have contributed to a conceptual design of an application, it is not yet implemented as a software app nor tested for its usability due to lack of funding.

Some researchers have not included people with impairments in the idea creation process, but first included them as participants in the evaluation of the digital health solution [38] (motor impairment), [39, 48] (visual impairment)). Typically, the researchers first designed a prototype before they invited the participants to assess the prototype in one or more iterations. After the assessment the prototype was further developed to meet their needs before they re-evaluated the usability and accessibility of the solution.

Ferati, Babar et al. [43] differed from the other sources that described participatory design approaches to foster access and utilisation of digital health solutions for people with impairments. They included participants with visual, motor and cognitive impairments from idea creation to design and deployment of a smart shower, but unfortunately did not evaluate the usability of the solution.

In contrast to the sources presented above that reported use of participatory design strategies to develop a specific digital health solution, three of the sources focused on strategies for ensuring the accessibility and usability of digital health solutions in general for people with impairments. To enhance accessibility and usability for people with visual impairment, one of the studies developed a set of general suggestions for designers and developers of home automation and remote-control systems [50]. The general suggestions were based on feedback from adults with visual impairments sharing needs, challenges and requests related to smart homes. The researchers suggested the development of one accessible and usable interface for all services, solutions that could be used with various services, functionalities that are customisable and possibilities to control the solution offline. Another study developed apps together with people with cognitive and communication impairments [52]. Based on experiences they suggested strategies, such as, appropriate language levels, making information accessible, adapt tools to match the participants’ cognitive needs to involve people with cognitive and communication impairment in mobile health app design. In addition, the researchers emphasised that communication aids, signs and gesture systems and observation of participant behaviour during the design process can be used to elicit participants’ perspectives. The last of the studies developed design guidelines to make apps in general accessible for people with visual impairment [51]. The guidelines contain several concrete suggestions, such as support for zoom in/out for the main content and other colour schemes, intuitive navigation and menu, highly legible fonts, etc. The researchers did not include people with visual impairments in the development phase, but people with and without visual impairments tested whether the guidelines made health apps more accessible. This was done by applying the guidelines on five randomly selected health apps. The study confirmed an increase in the actual information recognition speed for people with and without visual impairment after applying the design guidelines to the five selected health apps [51].

## Discussion

To the best of our knowledge, this is the first scoping review that investigates the experiences, needs and barriers related to the use of digital health solutions and to participation by people with impairments from a disease-agnostic perspective, starting from idea to design and deployment, for improved accessibility and utilisation of digital health solutions. In line with previous research [9–13], this scoping review shows that digital health solutions are often designed in ways that make them inaccessible or at best challenging to use for people with impairments. The review also showed that people with impairments request digital health solutions to accommodate their specific impairment-related challenges and that are also easy and intuitive to use, which is also consistent with previous research [10, 13]. Overall, the large number of articles that were excluded from this scoping review, strengthens the impression that digital health solutions often do not come with designs that cater for the diverse needs of people with different types of impairments. This finding is of concern for public health, as a lack of attention to the needs of people with impairments can lead to increased health inequality, leaving people with impairments unable to take full advantage of digital health solutions.

This scoping review showed that people with impairments reported a range of technological design needs and barriers related to perceivable content (use of colour, contrast, resize text), operability, navigation, input modalities, understandability and predictability. Several of these needs and barriers are in line with WCAG guidelines [5]. While conformance to WCAG is an important first step towards creating solutions that are more usable and accessible for people with and without impairments [53], it does not guarantee universal design [54, 55]. Therefore, universal design efforts should be based on a human-centred and participatory design process, involving a broad range of stakeholders throughout the design process, including people with impairments. This scoping review affirms that when the people with impairments were included from the idea creation phase and throughout the development of digital health solutions, the expressed accessibility and usability of the developed solution is good. This finding indicates that it is paramount to ensure involvement during the whole design process to ensure that digital health solutions are capable to mitigate the specific impairment-related challenges that prohibit people with impairments from using digital health applications.. Early inclusion may also contribute to reducing structural inequalities and promoting health equity through use value and availability of digital health solutions.

Overall, this scoping review indicates that knowledge about the general principles of universal design should be used in the development of digital health solutions. The review shows that several of the barriers reported by persons with impairments could be reduced if principles in WCAG were utilised. Instead of focusing on achieving universal design, the included sources focused on digital health solutions that are designed to meet the needs of one specific user group, for example, people with motor impairment or cognitive impairment. The focus might be explained by the inherent heterogeneity, that people with different types of impairments experience different challenges related to digital health solutions [10, 13]. However, people with a certain type of impairment may also have other types of impairments or age-related challenges. Therefore, basing health solutions on universal design principles will make them usable and accessible to wider user groups and thus make them more sustainable.

Drawing from and in accordance with the ICF definition, an impairment does not necessarily imply a disability or disadvantage if the personal and external factors that represent the circumstances in the person’s life are adapted to the person’s capacity and health condition [2, 4]. Disability can occur when external factors such as digital health solutions do not meet people’s needs, context and state of health. Lack of adherence to universal design principles can create disability or disadvantage. A core objective is that digital health solutions are designed to support peoples’ health management and their quality of life, foster engagement and empower them. However, previous research [9–14] included in this scoping review indicates that digital health solutions are (too) often inaccessible, because they fail to consider the relationship between the user, their context and the digital health solution. Thus, the solution does not support their health management and quality of life.

Furthermore, this review indicates that a low degree of user participation of people with impairments early in the development of digital health solutions may in fact introduce additional barriers and exclusion, as people with impairments do not fully benefit from the deployed solutions. In other words, if the development of digital health solutions do not apply participatory and universal design, the digital solution is less likely to be adapted to people’s state of health, permanent or transient impairment and capacity to use. For public health this may actually cause a situation when people who are normally considered fully abled suddenly appear disabled. This argument is in line with previous research [56–58], which emphasises that digital health solutions with “one-for-all” design can drive increased health inequalities because heterogeneity is not well incorporated.

### Strengths and limitations

The inclusion criterions may have led to knowledge about participation in design, access or utilisation not being included in this scoping review if the sources have not clearly described that the knowledge is based on the perspectives of people with impairments. An obvious strength in taking a disease-agnostic stance allowed us to focus on functional impairments that could, if accounted for, ease user experiences with digital solutions. One limitation is that due to language barriers, only literature written in Norwegian, Swedish, Danish and English were included. Another limitation is that relevant research conducted before 2015 has not been included due to the recent development of digital health solutions. This review may not have identified all published literature despite attempts to include a range of keywords and databases to be as comprehensive as possible. A limitation may also be that we retained literature for further analysis if it clearly included and reported the perspective of people with impairments. However, as explained in the results section, several of the excluded sources stated that they had a participatory design, but the perspective of people with impairment was not accounted for.

## Conclusion

According to the internationally accepted convention CRPD [18], digital health solutions must be accessible and usable for all, including people with impairments and other underserved groups. This scoping review indicates that the use of universal design principles and actively engaging with people having different types of permanent or transient impairments, starting in the idea creation phase of digital health solutions, is more likely to result in solutions that are accessible to everyone regardless of their state of health. Since this scoping review shows that available digital health solutions often are inaccessible for people with impairments, there is a strong need to build capacity, also in public health, to meet the requirement for design of solutions that follow universal design principles, to meet people’s needs, context and state of health. The large number of inaccessible solutions currently available is likely to maintain patterns of systemic exclusion of people with impairments, which comes with significant implications for public health efforts. In fact, WHO states that digital health is vital for health and wellbeing, and in achieving universal health coverage [1]. With aging populations, the proportion of people with disabilities and comorbidities are increasing. This does not only lead to an increased demand for digital health services, but more specifically it leads to an increased demand for accessible and universally designed solutions used as part of the health services. Although we acknowledge desirability and challenges to develop digital health solutions that adapt to the diversity of the human body function and structure, peoples’ capabilities and their context, a thorough discussion of possibilities and efforts needed to create accessible digital solutions is overdue.

## Supplementary Information

**Additional file 1.**

**Additional file 2.**

**Additional file 3.**

## Data Availability

All data generated or analysed during this study are included in this published article and its supplementary information files (Appendix 2 and 3).

## Abbreviations

ICF: International Classification of Functioning Disability and Health
WCAG: Web Content Accessibility Guidelines
WHO: World Health Organization
CRPD: Convention on the Rights of Persons with Disabilities
