# Supplementary material for: The experiences, needs and barriers of people with impairments related to usability and accessibility of digital health solutions, levels of involvement in the design process and strategies for participatory and universal design: a scoping review
Source: BMC Public Health. 2022 Jan 6;22:35. doi: 10.1186/s12889-021-12393-1 (PMC8734131; doi:10.1186/s12889-021-12393-1)
Supplement: Supplementary file 2 — Additional file 2. [file 12889_2021_12393_MOESM2_ESM.docx]

**Appendix 2**

**Database: MEDLINE (Ovid)**

Date of search: 05.05.2020
Number of hits: 1453

1 disabled persons/ or Intellectual disability/ or disabled children/ or mentally disabled persons/

2 persons with hearing impairments/ or Visually impaired persons/ or Blindness/ or Hearing loss/ or deafness/

3 (disabilit* or disabled or impaired or impairment* or handicap* or blind or blindness or hearing loss or deafness or deaf).tw,kf.

4 ((cognitive or mental* or psychological or physical* or physiological or function* or visual* or hearing or sensor* or language or motor*) adj2 (decline* or deficit*)).tw,kf.

5 or/1-4

6 (ubiquitous health or uhealth or u-health or m-health or mhealth or mobile health* or imhere or mobile rehabilitation or mrehab).tw,kf.

7 telemedicine/ or telerehabilitation/ or (telemedicine or tele-medicine or telemedical or tele-medical or telecare or tele-care or telehealth or tele-health or teletreatment* or tele-treatment* or telerehabilitation or tele-rehabilitation or ehealth or e-health or electronic health).tw,kf.

8 Internet-based intervention/ or ((web based or internet based or computer based) adj2 intervention*).tw,kf.

9 (emental or e-mental or eMediplan or emedication* or e-medication*).tw,kf.

10 health records, personal/ or ((personal* or patient*) adj3 (health Record* or portal*)).tw,kf.

11 Remote Consultation/ or (teleconsultation* or tele-consultation* or econsultation* or e-consultation* or (video adj2 consultation*) or (online adj2 consultation*) or (on-line adj2 consultation*) or (remote adj2 consultation*)).tw,kf.

12 ((digital adj2 service*) or (augmented reality or ambient assisted living* or ambient intelligence*)).tw,kf.

13 ((assist* technolog* or self help or self management or self monitor* or self care or self medication*) and (digital or online or internet or web or electronic or application* or app or apps or tablet*)).tw,kf.

14 Self-Help Devices/ and (digital or online or internet or web or electronic or application* or app or apps or tablet*).tw,kf.

15 ((digital or mobile*) adj2 sensor*).tw,kf.

16 ((welfare adj2 technolog*) or smarthome* or smart home*).tw,kf.

17 (((digital or information or communication) adj2 technolog*) and (service* or health* or care)).tw,kf.

18 ((health* or education*) adj4 (digital or online or internet or web or electronic or application* or app or apps or tablet*)).tw,kf.

19 (patient education and (digital or online or internet or web or electronic or application* or app or apps or tablet*)).tw,kf.

20 ((game* or gaming or virtual reality) adj3 (training or exercise* or rehabilitation or interactive)).tw,kf.

21 (Video Games/ and Exercise/) or exergame*.tw,kf.

22 or/6-21

23 Health services accessibility/ or communication barriers/ or digital divide/ or (barrier* or facilitator* or digital divide*).tw,kf.

24 "treatment adherence and compliance"/ or "patient acceptance of health care"/ or patient compliance/ or medication adherence/ or patient dropouts/ or patient participation/ or medication adherence.tw,kf.

25 patient satisfaction/ or patient preference/

26 ((patient* or consumer* or user* or client* or participant*) adj3 (satisfaction* or participat* or innovat* or preference* or compliance* or adherence* or nonadherence or involvement* or perspective* or experience* or view* or need*)).tw,kf.

27 Decision Making/ or (decision making or shared decision*).tw,kf.

28 environment design/ or universal design/ or design*.ti,kf.

29 ((cocreat* or co-creat* or participator* or universal or inclusive) adj4 design*).tw,kf.

30 Healthcare Disparities/ or Health Equity/ or ((disparit* or inequalit* or equit*) and health*).tw,kf.

31 (social inclusion* or social exclusion* or universal access).tw,kf.

32 computer literacy/ or Health Literacy/ or literacy.tw,kf.

33 ((inclusive or identity or person first) adj language).tw,kf.

34 empowerment/ or empower*.tw,kf.

35 Patient-Centered Care/ or (patient cent* or person cent* or user cent*).tw,kf.

36 (technolog* adj2 (readiness or capab*)).tw,kf.

37 "Social Determinants of Health"/ or social determinant*.tw,kf.

38 ((digital adj2 inclusion*) or (digital adj2 exclusion*)).tw,kf.

39 ((patient* or consumer* or user* or client* or participant*) adj4 (utilit* or accept* or useful* or accessib*)).tw,kf.

40 (utilit* or accept* or useful* or accessib*).ti,kf.

41 (usab* or understandab* or learnab* or operab* or attractive* or user friendl*).tw,kf.

42 or/23-41

43 5 and 22 and 42

44 limit 43 to (yr="2015 -Current" and (danish or english or norwegian or swedish))

**Database: CINAHL (Ebsco)**Date of search: 05.05.2020
Number of hits: 970

S1 (MH "Disabled+") OR (MH "Amputees") OR (MH "Athletes, Disabled") OR (MH "Child, Disabled") OR (MH "Mentally Disabled Persons") OR (MH "Parents, Disabled") OR (MH "Students, Disabled") OR (MH "Intellectual Disability+") OR (MH "Developmental Disabilities") OR (MH "Blindness")

S2 TI (cognitive OR mental* OR psychological OR physical* OR physiological OR function* OR visual* OR hearing OR sensor* OR language OR motor*) N1 (decline* OR deficit*) OR AB (cognitive OR mental* OR psychological OR physical* OR physiological OR function* OR visual* OR hearing OR sensor* OR language OR motor*) N1 (decline* OR deficit*)

S3 (blind OR blindness OR "hearing loss" OR deafness OR deaf OR disabilit* OR disabled OR impaired OR impairment* OR handicap*)

S4 S1 OR S2 OR S3

S5 TI ( "ubiquitous health" OR uhealth OR "u health" OR "m health" OR mhealth OR "mobile health*" OR imhere OR "mobile rehabilitation" OR mrehab ) OR AB ( "ubiquitous health" OR uhealth OR "u health" OR "m health" OR mhealth OR "mobile health*" OR imhere OR "mobile rehabilitation" OR mrehab )

S6 (MH "Telehealth+") OR (MH "Telemedicine+") OR (MH "Remote Consultation") OR (MH "Telerehabilitation") OR (MH "Telenursing") OR (MH "Telepsychiatry")

S7 TI ( telemedicine OR "tele medicine" OR telecare OR "tele care" OR telehealth OR "tele health" OR telemedical OR "tele medical" OR teletreatment* OR "tele treatment*" OR telerehabilitation OR "tele rehabilitation" OR ehealth OR "e health" OR "electronic health" ) OR AB ( telemedicine OR "tele medicine" OR telecare OR "tele care" OR telehealth OR "tele health" OR teletreatment* OR "tele treatment*" OR telerehabilitation OR "tele rehabilitation" OR ehealth OR "e health" OR "electronic health" )

S8 TI ( ("web based" OR "internet based" OR "computer based") N1 intervention* ) OR AB ( ("web based" OR "internet based" OR "computer based") N1 intervention* )

S9 TI ( emental OR "e mental" OR eMediplan OR emedication* OR "e medication*" ) OR AB ( emental OR "e mental" OR eMediplan OR emedication* OR "e medication*"

S10 (MH "Patient Portals") OR (MH "Medical Records, Personal")

S11 TI ( (personal* OR patient*) N2 ("health record*" OR portal*) ) OR AB ( (personal* OR patient*) N2 ("health record*" OR portal*) )

S12 (MH "Remote Consultation") OR TI (( teleconsultation* OR "tele consultation*" OR econsultation* OR "e consultation*" OR (video N1 consultation*) OR (online N1 consultation*) OR ("on line" N1 consultation*) OR (remote N1 consultation*) ) OR AB ( teleconsultation* OR "tele consultation*" OR econsultation* OR "e consultation*" OR (video N1 consultation*) OR (online N1 consultation*) OR ("on line" N1 consultation*) OR (remote N1 consultation*) ))

S13 TI digital N1 service* OR AB digital N1 service*

S14 TI "augmented reality" OR AB "augmented reality"

S15 TI ( "ambient assisted living*" OR "ambient intelligence*" ) OR AB ( "ambient assisted living*" OR "ambient intelligence*" )

S16 TI ( ((“assist* technolog*” OR “self help” OR “self management” OR “self monitor*” OR “self care” OR “self medication*”) AND (digital OR online OR internet OR web OR electronic OR application* OR app OR apps OR tablet*)) ) OR AB ( ((“assist* technolog*” OR “self help” OR “self management” OR “self monitor*” OR “self care” OR “self medication*”) AND (digital OR online OR internet OR web OR electronic OR application* OR app OR apps OR tablet*)) )

S17 TI(digital OR online OR internet OR web OR electronic OR application* OR app OR apps OR tablet*) AND MH "Assistive Technology Devices+" ) OR ( AB(digital OR online OR internet OR web OR electronic OR application* OR app OR apps OR tablet*) AND MH "Assistive Technology Devices+" )

S18 TI ( (digital or mobile*) N1 (sensor or sensors) ) OR AB ( (digital or mobile*) N1 (sensor or sensors) )

S19 TI ( (welfare N1 technolog*) OR smarthome* OR "smart home*" ) OR AB ( (welfare N1 technolog*) OR smarthome* OR "smart home*" )

S20 TI ( (digital or information or communication*) N1 technolog*) AND (service* or health* or care) ) OR AB ( (digital or information or communication*) N1 technolog*) AND (service* or health* or care) )

S21 TI ( (health* OR education*) N3 (digital OR online OR internet OR web OR electronic OR application* OR app OR apps OR tablet*) ) OR AB ( (health* OR education*) N3 (digital OR online OR internet OR web OR electronic OR application* OR app OR apps OR tablet*) )
S22 TI ( "patient education" AND (digital OR online OR internet OR web OR electronic OR application* OR app OR apps OR tablet*) ) OR AB ( "patient education" AND (digital OR online OR internet OR web OR electronic OR application* OR app OR apps OR tablet*) )

S23 TI ( (game* OR gaming OR “virtual reality”) N2 (training OR exercise* OR rehabilitation OR interactive) ) OR AB ( (game* OR gaming OR “virtual reality”) N2 (training OR exercise* OR rehabilitation OR interactive )

S24 ((MH "Video Games+") OR (MH "Games+")) AND (MH "Exercise+")

S25 (MH "Exergames")

S26 S5 OR S6 OR S7 OR S8 OR S9 OR S10 OR S11 OR S12 OR S13 OR S14 OR S15 OR S16 OR S17 OR S18 OR S19 OR S20 OR S21 OR S22 OR S23 OR S24 OR S25

S27 S4 AND S26

S28 (MH "Health Services Accessibility+") OR (MH "Healthcare Disparities")

S29 (MH "Communication Barriers") OR (MH "Digital Divide") OR barrier* OR facilitator* OR "digital divide*")

S30 (MH "Medication Compliance") OR (MH "Treatment Refusal") OR (MH "Consumer Participation") OR (MH "Patient Satisfaction") OR (MH "Patient Preference") OR (MH "Consumer Satisfaction") OR TI "medication adherence" OR AB "medication adherence"
S31 TI ( (patient* OR consumer* OR user* OR client* OR participant*) N2 (satisfaction* OR participat* OR innovat* OR preference* OR compliance* OR adherence* OR nonadherence OR involvement* OR perspective* OR experience* OR view* OR need*) ) OR AB ( (patient* OR consumer* OR user* OR client* OR participant*) N2 (satisfaction* OR participat* OR innovat* OR preference* OR compliance* OR adherence* OR nonadherence OR involvement* OR perspective* OR experience* OR view* OR need*) )

S32 (MH "Decision Making, Patient") OR (MH "Decision Making, Shared") OR (MH "Decision Making")

S33 TI ( "decision making" OR "shared decision*" ) OR AB ( "decision making" OR "shared decision*" )

S34 TI ( (cocreat* OR "co-creat*" OR participat* OR universal OR inclusive) N3 design* ) OR AB ( (cocreat* OR "co-creat*" OR participat* OR universal OR inclusive) N3 design*

S35 (MH "Systems Development+") OR TI design*

S36 (MH "Health Services Needs and Demand")

S37 TI ( (disparit* OR inequalit* OR equit*) AND health* ) OR AB ( (disparit* OR inequalit* OR equit*) AND health* )

S38 TI ( "social inclusion*" OR "social exclusion*" OR "universal access" ) OR AB ( "social inclusion*" OR "social exclusion*" OR "universal access" )

S39 (MH "Information Literacy") OR (MH "Health Literacy")

S40 TI literacy OR AB literacy

S41 TI ( "inclusive language" OR "identity language" OR "person first language" ) OR AB ( "inclusive language" OR "identity language" OR "person first language" )

S42 (MH "Empowerment") OR TI empower* OR AB empower*

S43 (MH "Patient Centered Care") OR TI ( "patient cent*" OR "person cent*" OR "user cent*" ) OR AB ( "patient cent*" OR "person cent*" OR "user cent*" )

S44 TI ( technolog* N1 (readiness or capab*) ) OR AB ( technolog* N1 (readiness or capab*) )

S45 MH "Social Determinants of Health" OR TI "social determinant*" OR AB "social determinant*"

S46 TI ( (digital N1 inclusion*) OR (digital N1 exclusion*) ) OR AB ( (digital N1 inclusion*) OR (digital N1 exclusion*) )

S47 TI (patient* OR consumer* OR user* OR client* OR participant*) N3 (utilit* OR accept* OR useful* OR accessib*) OR AB (patient* OR consumer* OR user* OR client* OR participant*) N3 (utilit* OR accept* OR useful* OR accessib*)

S48 TI utilit* OR accept* OR useful* OR accessib*

S49 TI ( usab* OR understandab* OR learnab* OR operab* OR attractive* OR “user friendl*” ) OR AB ( usab* OR understandab* OR learnab* OR operab* OR attractive* OR “user friendl*” )

S50 S28 OR S29 OR S30 OR S31 OR S32 OR S33 OR S34 OR S35 OR S36 OR S37 OR S38 OR S39 OR S40 OR S41 OR S42 OR S43 OR S44 OR S45 OR S46 OR S47 OR S48 OR S49

S51 S4 AND S26 AND S50 Limiters - Published Date: 20150101-20200631; Peer Reviewed; Language: Danish, English, Norwegian, Swedish

**Database: Scopus (Elsevier)**

Date of search: 08.05.2020
Number of hits: 2720

(TITLE-ABS-KEY ( disabilit* OR disabled OR impaired OR impairment* OR handicap* OR blind OR blindness OR "hearing loss" OR deafness OR deaf OR ( ( cognitive OR mental* OR psychological OR physical* OR physiological OR function* OR visual* OR hearing OR sensor* OR language OR motor* ) W/1 ( decline* OR deficit* ) ) )) AND ((TITLE-ABS-KEY ( "ubiquitous health" OR uhealth OR "u-health" OR "m-health" OR mhealth OR "mobile health*" OR imhere OR "mobile rehabilitation" OR mrehab OR telemedicine OR telemedical OR "tele-medical" OR "tele-medicine" OR telecare OR "tele-care" OR telehealth OR "tele-health" OR teletreatment* OR "tele-treatment*" OR telerehabilitation OR "tele-rehabilitation" OR ehealth OR "e-health" OR "electronic health" OR emental OR "e-mental" OR eMediplan OR emedication* OR "e-medication*")) OR (TITLE-ABS-KEY ( ( "web based" OR "internet based" OR "computer based" ) W/1 intervention* )) OR (TITLE-ABS-KEY((personal* W/1 "health record*") OR (personal* W/1 portal*) OR (patient* W/1 "health record*") OR (patient* W/1 portal*))) OR (TITLE-ABS-KEY(teleconsultation* OR "tele-consultation*" OR econsultation* OR "e-consultation*" OR (video w/1 consultation*) OR (online w/1 consultation*) OR ("on-line" w/1 consultation*) OR (remote w/1 consultation*))) OR (TITLE-ABS-KEY((digital w/1 service*) OR "augmented reality" OR "ambient assisted living*" OR "ambient intelligence*")) OR (TITLE-ABS-KEY("assist* technolog*" OR "self help" OR "self management" OR "self monitor*" OR "self care" OR "self medication*") W/4 (digital OR online OR internet OR web OR electronic OR application* OR app OR apps OR tablet*)) OR (TITLE-ABS-KEY((digital W/1 sensor*) OR ( mobile* W/1 sensor*) OR (welfare W/1 technolog*) OR smarthome* OR "smart home*")) OR (TITLE-ABS-KEY((digital W/1 technolog*) OR (information W/1 technolog*) OR (communication W/1 technolog*)) AND (service* OR health* OR care)) OR (TITLE-ABS-KEY((health* OR education*) W/2 (digital OR online OR internet OR web OR electronic OR application* OR app OR apps OR tablet*))) OR (TITLE-ABS-KEY("patient education" AND (digital OR online OR internet OR web OR electronic OR application* OR app OR apps OR tablet*))) OR (TITLE-ABS-KEY(exergame* OR ((game* or gaming or "virtual reality") W/2 (training or exercise* or rehabilitation or interactive))))) AND (((TITLE-ABS-KEY(barrier* OR facilitator* OR "digital divide*" OR "medication adherence*")) OR (TITLE-ABS-KEY((patient* OR consumer* OR user* OR client* OR participant*) W/2 (satisfaction* OR participat* OR innovat* OR preference* OR compliance* OR adherence* OR nonadherence OR involvement* OR perspective* OR experience* OR view* OR need*))) OR (TITLE-ABS-KEY("decision making" OR "shared decision*" OR "environment design" OR "universal design" OR ((cocreat* OR "co-creat*" OR participator* OR universal OR inclusive) W/2 design*))) OR (TITLE(design*)) OR (TITLE-ABS-KEY((disparit* OR inequalit* OR equit*) AND health*)) OR (TITLE-ABS-KEY("social inclusion*" OR "social exclusion*" OR "universal access" OR literacy)) OR (TITLE-ABS-KEY("inclusive language" OR "identity language" OR "person first language")) OR (TITLE-ABS-KEY("patient cent*" OR "person cent*" OR "user cent*" OR empower* OR "social determinant*")) OR (TITLE-ABS-KEY((digital W/1 inclusion*) OR (digital W/1 exclusion*))) OR (TITLE-ABS-KEY(technolog* W/1 (readiness OR capab*))) OR (TITLE-ABS-KEY("health services accessibility"))) OR ((TITLE-ABS-KEY((patient* OR consumer* OR user* OR client* OR participant*) W/2 (utilit* OR accept* OR useful* OR accessib*)) OR TITLE((utilit* OR accept* OR useful* OR accessib*) ) OR TITLE-ABS-KEY((usab* OR understandab* OR learnab* OR operab* OR attractive* OR "user friendl*"))))) AND ( LIMIT-TO ( DOCTYPE,"ar" ) OR LIMIT-TO ( DOCTYPE,"cp" ) OR LIMIT-TO ( DOCTYPE,"re" ) OR LIMIT-TO ( DOCTYPE,"cr" ) ) AND ( LIMIT-TO ( PUBYEAR,2020) OR LIMIT-TO ( PUBYEAR,2019) OR LIMIT-TO ( PUBYEAR,2018) OR LIMIT-TO ( PUBYEAR,2017) OR LIMIT-TO ( PUBYEAR,2016) OR LIMIT-TO ( PUBYEAR,2015) ) AND ( LIMIT-TO ( LANGUAGE,"English" ) )

**Database: ACM Library**

Date of search: 08.05.2020
Number of hits: 710

Search 1:

(impair* OR disab* OR blind OR blindness OR "hearing loss" OR deafness OR deaf OR handicap* OR ((cognitive OR mental* OR psychological OR physical* OR physiological OR function* OR visual* OR hearing OR sensor* OR language OR motor*) AND (decline* OR deficit*)))

Search 2:
“ubiquitous health” OR uhealth OR “u-health” OR “m-health” OR mhealth OR “mobile health*” OR imhere OR “mobile rehabilitation” OR mrehab OR telemedicine OR “tele-medicine” OR telemedical OR telecare OR “tele-care” OR telehealth OR “tele-health” OR teletreatment* OR “tele-treatment*” OR telerehabilitation OR “tele-rehabilitation” OR ehealth OR “e-health” OR “electronic health” OR “Internet based intervention” OR “web based intervention*” OR “computer based intervention*” OR “personal portal*” OR “personal* health record*” OR “personal health portal*” OR “patient health record*” OR “patient* portal*” OR “patient* health portal*” OR emental OR “e-mental” OR eMediplan OR emedication* OR “e medication*” OR teleconsultation* OR "tele consultation*" OR econsultation* OR "e consultation*" OR “video consultation*” OR “online consultation*” OR "on-line consultation*” OR “remote consultation*” OR “digital health service*” OR “augmented reality” OR “ambient assisted living*” OR “ambient intelligence*” OR “digital sensor*” OR “mobile sensor*” OR “welfare technolog*” OR smarthome* OR “smart home*” OR exergame* OR “assist* technolog*” OR “digital technolog*” OR “information technolog*” OR “communication technolog*” OR game* OR gaming OR “virtual reality”

Search 3:
(“digital divide*” OR barrier* OR facilitator* OR “patient acceptance of health care" OR “medication adherence” OR ((patient* OR consumer* OR user* OR client* OR participant*) AND (satisfaction* OR participat* OR innovat* OR preference* OR compliance* OR adherence* OR nonadherence OR involvement* OR perspective* OR experience* OR view* OR need*)) OR “decision making” OR “shared decision*” OR ((cocreat* OR “co-creat*” OR participator* OR universal OR inclusive) AND design*)) OR ((disparit* OR inequalit* OR equit*) AND health*)) OR “social inclusion*” OR “social exclusion*” OR “universal access” OR literacy OR “inclusive language” OR “identity language” OR “person first language” OR empower* OR “patient cent*” OR “person cent*” OR “user cent*” OR “technolog* readiness” OR “technolog* capab*” OR “social determinant*” OR “digital inclusion*” OR “digital exclusion*” OR utilit* OR accept* OR useful* OR accessib* OR usab* OR understandab* OR learnab* OR operab* OR attractive* OR “user friendl*”)

Searched with different combinations with regard to which fields have been searched.
search 1:title AND search 2:all fields AND search 3:title
search 1: abstract AND search 2: all fields AND search 3: title
search 1: title AND search l 2: all fields AND search 3: keyword
search 1: abstract AND search 2: all fields AND search 3: keyword

**Database: IEE Explore**

Date of search: 09.05.2020
Number of hits: 113

Search 1:

("Document Title": utility OR "Document Title": acceptance OR "Document Title": usefulness OR "Document Title": usable OR "Document Title": usability OR "Document Title": Accessibility OR "Document Title": satisfaction OR "Document Title": participation OR "Document Title": preference OR "Document Title": preferences OR "Document Title": compliance OR "Document Title": adherence OR "Document Title": involvement OR "Document Title": involved OR "Document Title": perspective OR "Document Title": perspectives OR "Document Title": experience OR "Document Title": experiences OR "Document Title": view OR "Document Title": views OR "Document Title": user design OR "Document Title": responsive design OR "Document Title": participatory design OR "Document Title": inclusive design OR "Document Title": universal design OR "Document Title": ux design OR "Document Title": understandable OR "Document Title": learnability OR "Document Title": operability OR "Document Title": “user friendly”) AND ("Index Terms": disab* OR "Index Terms": impairment* OR "Index Terms": handicap*) AND ("Index Terms": healthcare OR "Index Terms": health OR "Index Terms": rehabilitation OR "Index Terms": service OR "Index Terms": services)

Search 2:

("Index Terms": “communication barriers” OR "Index Terms": “digital divide” OR "Index Terms": “digital divides” OR "Index Terms": “patient compliance” OR "Index Terms": “medication adherence” OR "Index Terms": “patient participation” OR "Index Terms": “patient satisfaction” OR "Index Terms": “patient preference” OR "Index Terms": “decision making” OR "Index Terms": “shared decision” OR
"Index Terms": “universal design” OR "Index Terms": “inclusive design” OR "Index Terms": “participatory design” OR "Index Terms": “health disparities” OR "Index Terms": “health inequalities” OR "Index Terms": “universal design” OR "Index Terms": “inclusive design” OR "Index Terms": “participatory design” OR "Index Terms": “health disparities” OR "Index Terms": “health inequalities” OR "Index Terms": “social inclusion” OR "Index Terms": “social exclusion” OR "Index Terms": “universal access” OR "Index Terms": “health Literacy” OR "Index Terms": “inclusive language” OR "Index Terms": “identity language” OR "Index Terms": “person first language” OR "Index Terms": empowerment OR "Index Terms": “patient centered” OR "Index Terms": “patient centred” OR "Index Terms": “person centered” OR "Index Terms": “person centered” OR "Index Terms": “user centered” OR "Index Terms": “user centred” OR "Index Terms": “technology readiness” OR "Index Terms": “technology capability” OR "Index Terms": “social determinant” OR "Index Terms": “social determinants” OR "Index Terms": “digital inclusion” OR "Index Terms": “digital exclusion” OR "Index Terms": “digital inclusions” OR "Index Terms": “digital exclusions”) AND ("Index Terms": disab* OR "Index Terms": impairment* OR "Index Terms": handicap*) AND ("Index Terms": healthcare OR "Index Terms": health OR "Index Terms": rehabilitation OR "Index Terms": service OR "Index Terms": services)
